# Enhanced photocatalytic and electrochemical performance of TiO_2_-Fe_2_O_3_ nanocomposite: Its applications in dye decolorization and as supercapacitors

**DOI:** 10.1038/s41598-020-58110-7

**Published:** 2020-01-27

**Authors:** M. R. Anil Kumar, Buzuayehu Abebe, H. P. Nagaswarupa, H. C. Ananda Murthy, C. R. Ravikumar, Fedlu Kedir Sabir

**Affiliations:** 1 Research Centre, Department of Science, East West Institute of Technology, Bangalore, 560091 India; 2 Department of Applied Chemistry, School of Applied Natural Science, Adama Science and Technology University, Po Box 1888, Adama, Ethiopia; 3 PG Department of Chemistry, Davanagere University, Davanagere, 577001 India

**Keywords:** Environmental chemistry, Natural hazards

## Abstract

This work reveals a green combustion route for the synthesis of TiO_2_, Fe_2_O_3_ and TiO_2_-Fe_2_O_3_ nanocomposites as photocatalysts for decolorization of Titan Yellow (TY) and Methyl Orange (MO) dyes at room temperature in aqueous solution concentration of 20 ppm under UV-light irradiation. We observed that the TiO_2_-Fe_2_O_3_ nanocomposite shows superior photocatalytic activity for TY dye compared to pure TiO_2_ and Fe_2_O_3_. Rate constant (k) values of TiO_2_, Fe_2_O_3_ and TiO_2_–Fe_2_O_3_ for TY and MO are 0.0194, 0.0159, 0.04396 and 0.00931, 0.00772 0.0119 kmin^−1^ respectively. The surface area and pore volume of TiO_2_-Fe_2_O_3_ nanocomposite were found to be 71.56 m^2^/g and 0.076 cm^3^/g, respectively as revealed by BET studies. From the Barrett–Joyner–Halenda (BJH) plot, the mean pore diameter of TiO_2_-Fe_2_O_3_ nanoparticles was found to be 2.43 nm. Further, the TiO_2_-Fe_2_O_3_ nanocomposite showed good electrochemical behavior as an electrode material for supercapacitors when compared to pure TiO_2_ and Fe_2_O_3_ nanoparticles resulted in stable electrochemical performance with nearly 100% coulombic efficiency at a scan rate of 10 mV/s for 1000 cycles. Interestingly, the novelty of this work is that the designed supercapacitors showed stable electrochemical performance even at 1000^th^ cycle, which might be useful for rechargeable supercapacitor applications. The electrochemical properties of the nanocomposites were compared by the data obtained by cyclic voltammograms, charge-discharge tests and electrochemical impedance spectroscopic studies. These results demonstrated that the TiO_2_-Fe_2_O_3_ nanocomposite showed stable performance compared to TiO_2_ and Fe_2_O_3_ nanoparticles at current density of 5 Ag^−1^.

## Introduction

Recently electrochemical studies have gained significant attention due to energy and environment related issues. Since the discovery of TiO_2_ and its applications as photo-anode for battery, splitting of water, supercapacitor, dye removal etc^1^., many different metal oxides and electrodes have been explored to enhance the energy conversion efficiency. Amongst a variety of semiconductor metal oxides, hematite (α-Fe_2_O_3_) was found to be a good anode material for supercapacitor application, which could be attributed to its high solar-to-hydrogen efficiency, encouraging optical band gap (~2 eV), outstanding chemical strength as well as ease of availability in the nature. However, α-Fe_2_O_3_ exhibits low experimental performance compared with the theoretical values, which is possibly due to poor conducting and oxygen evolution properties in addition to short whole diffusion length. To overcome these problems, various synthetic methods have been tried in the recent past, to improve the experimental performance of α-Fe_2_O_3_.But, solution-based combustion method is a simple, low cost, energy saving, easy to control the surface morphology and particle size^2^. In addition, the materials prepared in this method are organic solvent free as we used water as solvent^3–5^.

The photocatalytic effectiveness of bare TiO_2_ is quiet less under direct solar light irradiation, despite of its superior physicochemical properties. This is possibly due to lack of visible light absorption as it exhibits high band gap energy (3.0–3.2 eV) and rapid electron-hole (e^−^/h^+^) recombination^6^. To reduce these drawbacks and improve structural stability, in the past several methods such as doping of hetero-junction with equivalent and/or different bandgap materials^7^, dyes sensitization^8^, noble and non-noble metal deposition^9^ were tried. Among those different methods explored, researchers tried to combine two or more semiconductor metal oxides having different band gaps^10–15^.

The present work was conducted on synthesis of TiO_2_, Fe_2_O_3_ and TiO_2_-Fe_2_O_3_ nanocomposites from Aloe Vera gel assisted green combustion method. The synthesized nanocomposites were characterized for morphological nature, structural feature, surface area, pore size, surface composition, particle size and band gap energies. The TiO_2_-Fe_2_O_3_composites offered improved photocatalytic efficiency for the decolorization of TY and MO dyes under the UV light irradiation compared with that of base TiO_2_ and Fe_2_O_3_ nano materials. In addition, TiO_2_-Fe_2_O_3_ nanocomposites have been investigated in a preliminary way for potential use as an electrode material for supercapacitor applications.

## Materials and Methods

### Synthesis of TiO_2_ and Fe_2_O_3_ nanocomposites (Green method)

TiO_2_ and Fe_2_O_3_ nanocomposites were synthesized by applying *Aloe Vera gel*^10^ as a fuel via solution combustion method. Freshly collected 20 ml of *Aloe Vera gel* was added to 80 ml of deionized water. The resulting solution was filtered to get gel. This gel was used as a fuel for the synthesis of TiO_2_ and Fe_2_O_3_ nanocomposites. The metal precursor salts of Titanium (IV) isopropoxide (Sigma Aldrich) and Ferric nitrate (Fe(NO_3_)_3_·9H_2_O) (Sigma Aldrich) were placed in two separate silica crucibles containing 5 ml of *Aloe Vera gel*. These mixtures were stirred using magnetic stirrer. The blends were placed in a pre-heated muffle furnace maintained at 380 ± 10 °C. The arrangement was bubbled to yield a transparent gel. The gel then formed a white froth, which extended to fill the vessel. From that point, the surface of the froth started burning and continued quickly all through the volume, leaving a white powder with a great degree of porous structure. The energy discharged from the response raised the temperature to 1200 °C which aided to shape TiO_2_ and Fe_2_O_3_ NPs.

### Synthesis of TiO_2_-Fe_2_O_3_ nanocomposite

To load iron oxides on titanium dioxide impregnation method was used with small modification like; replacement of water in place of ethanol and using simple stirrer in place of ultra-sonication^16^, which decreases cost and toxicities towards organic solvent. Having made those important modifications, 0.1 M of TiO_2_-Fe_2_O_3_ oxide materials were synthesized by adding appropriate amount of TiO_2_ andFe_2_O_3_ powder in aqueous solution, with continuous stirring at 80 °C. At this point, homogenous woody colored solution was observed. In addition, on drop wise addition of ammonia (NH_3_) to shift the pH of the solution to basic, the same color, but, a heterogeneous (fall apart) type solution was observed. After drying and grinding the obtained product was subjected to calcination at 500 °C for 3 hours.

### Characterization

The structural details of the nanocomposites were explored by using Shimadzu x-ray diffractometer (PXRD-7000) with CuKα (λ = 1.541 Ǻ). The vibrational spectra of the samples were recorded with a Shimadzu’s FTIR spectrophotometer (IR Affinity 1 S) using KBr pellets (400–4000 cm^−1^). The UV-Visible spectra of the samples were recorded in the range 200–800 nm using Shimadzu’s UV-2600, Uv-visible spectrophotometer. The morphological features and EDS analysis of all the samples were evaluated by using Field emission scanning electron microscope (FESEM/FIB-model Neon-40), at Nano manufacturing technology center (NMTC), CMTI, India. The Brunauer–Emmett–Teller (BET) method was utilized to calculate the specific surface areas of the samples in relative pressure (P/P_o_) range of 0.05–0.25. The pore-size distributions of the samples were determined by Barrett–Joyner–Halenda (BJH) method. The total pore volume of nanocomposites was accumulated at a relative pressure of P/P_o_ = 0.99. Sorption measurements of all the nanomaterials were carried out at −196 °C using a Quanta chrome instrument. The cyclic voltammetric studies were made using CHI604E potentiostat (tri-electrode system-sample as working electrode, platinum wire as counter electrode, and Ag/AgCl as reference electrode with 6.0 M KOH as electrolyte. The potential range utilized during these studies is ranging between −1.5 V and 0.9 V. The scanning has been carried out from 10 mV/s to50 mV/s. The potential range for recording the galvanostatic charge-discharge cycles is ranging between 0 and 0.7 V at a current density of 5 Ag^−1^. AC impedance measurements were carried out in the frequency range between 1 Hz and 1 MHz, with AC amplitude of 5 mV.

## Results and Discussions

**Figure 1:**
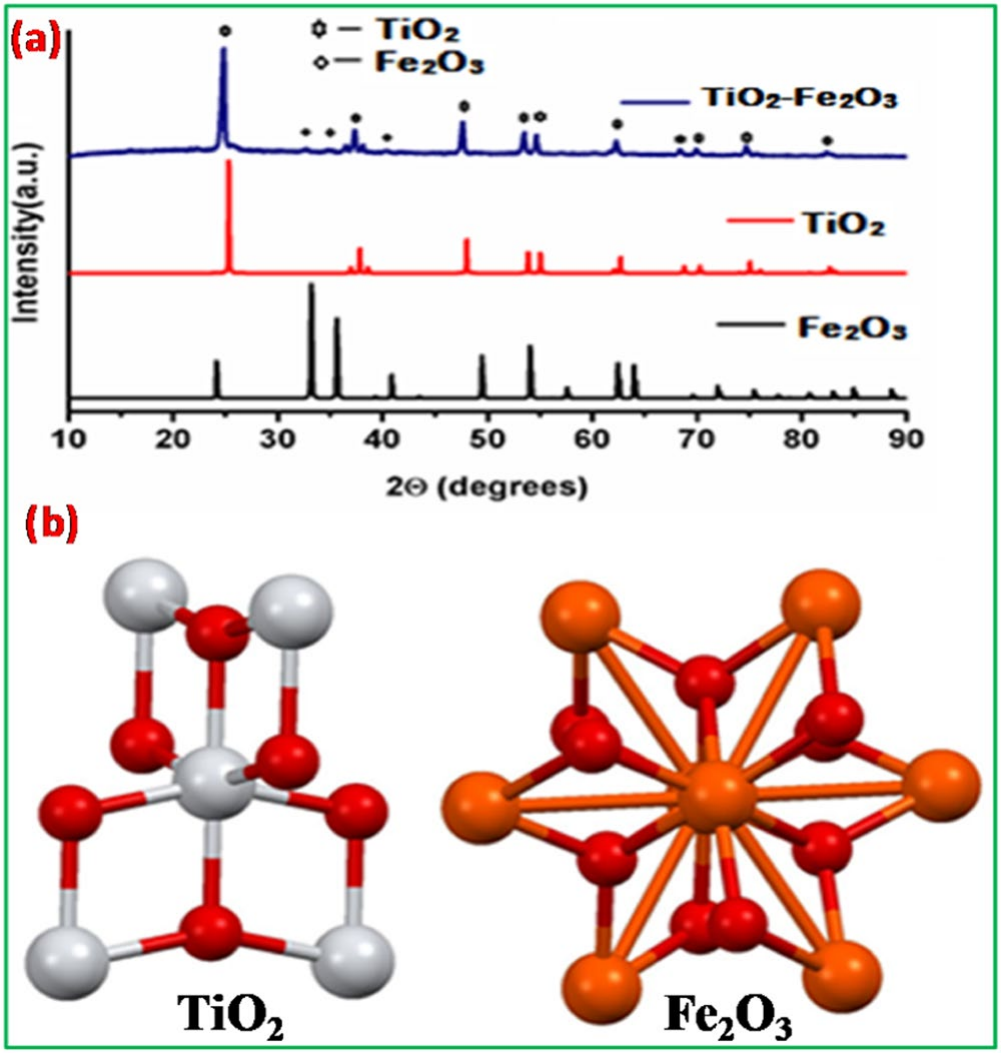
(**a**) PXRD pattern of TiO_2_, Fe_2_O_3_ and TiO_2_-Fe_2_O_3_ nano composites. **(b)** Structure of TiO_2_ and Fe_2_O_3_NPs in the nanocomposite.

As shown in Fig. 1(a) the composition and structure of synthesized materials were identified by PXRD spectra and is in agreement with tetragonal (anatase) and rhombohedral phases of TiO_2_ and Fe_2_O_3_ respectively. The 2θ and Miller indices matching to 25.2° (101), 37.3° (004), 49.2° (200), 53.9° (105), 54.7° (211), 63.1° (204), 74.8° (215) are assigned to the tetragonal anatase TiO_2_ were matched with JCPDS Card No. (21–1272) and 24^o^ (012), 33^o^ (104), 36^o^ (110), 41^o^ (113), 49^o^ (024), 54^o^ (115), 58^o^ (112), 63^o^ (214), 64^o^ (300) corresponds to Fe_2_O_3_ were well matched with JCPDS Card No. (00-001-1053). Confirming this, 33^o^ (104), 36^o^ (110) and 41^o^ (113) peaks of Fe_2_O_3_ were observed on the spectra of TiO_2_-Fe_2_O_3_ heterojunction^17–21^. The structural drawings obtained from exploration and analysis software for TiO_2_ and Fe_2_O_3_ mercury crystal structure visualization are show in Fig. 1(b).

**Figure 2:**
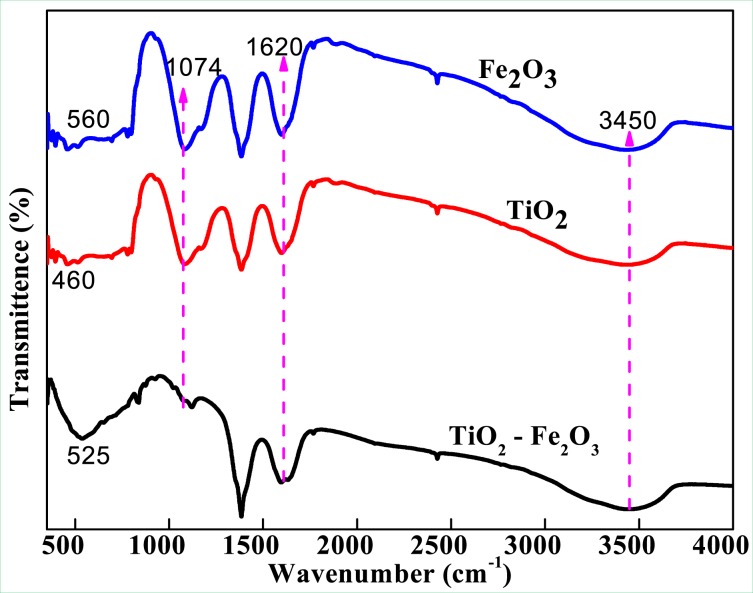
FTIR spectra of TiO_2_-Fe_2_O_3_ nanocomposites.

FTIR spectra of synthesized nanocomposite (TiO_2_-Fe_2_O_3_), recorded in the range of 400–4000 cm^−1^ are shown in the Fig. 2. The peak patterns appeared in the regions of 1074 cm^−1^, 1620 cm^−1^ and 3450 cm^−1^ corresponds to TiO_2_-Fe_2_O_3_ nanocomposite. The reduction in the intensity of all the peaks was observed in case of composites after the incorporation of Fe_2_O_3_. The broad peaks appeared at 3450 cm^−1^ and 1620 cm^−1^ corresponds to –OH stretching and bending vibrations^22^.

**Figure 3:**
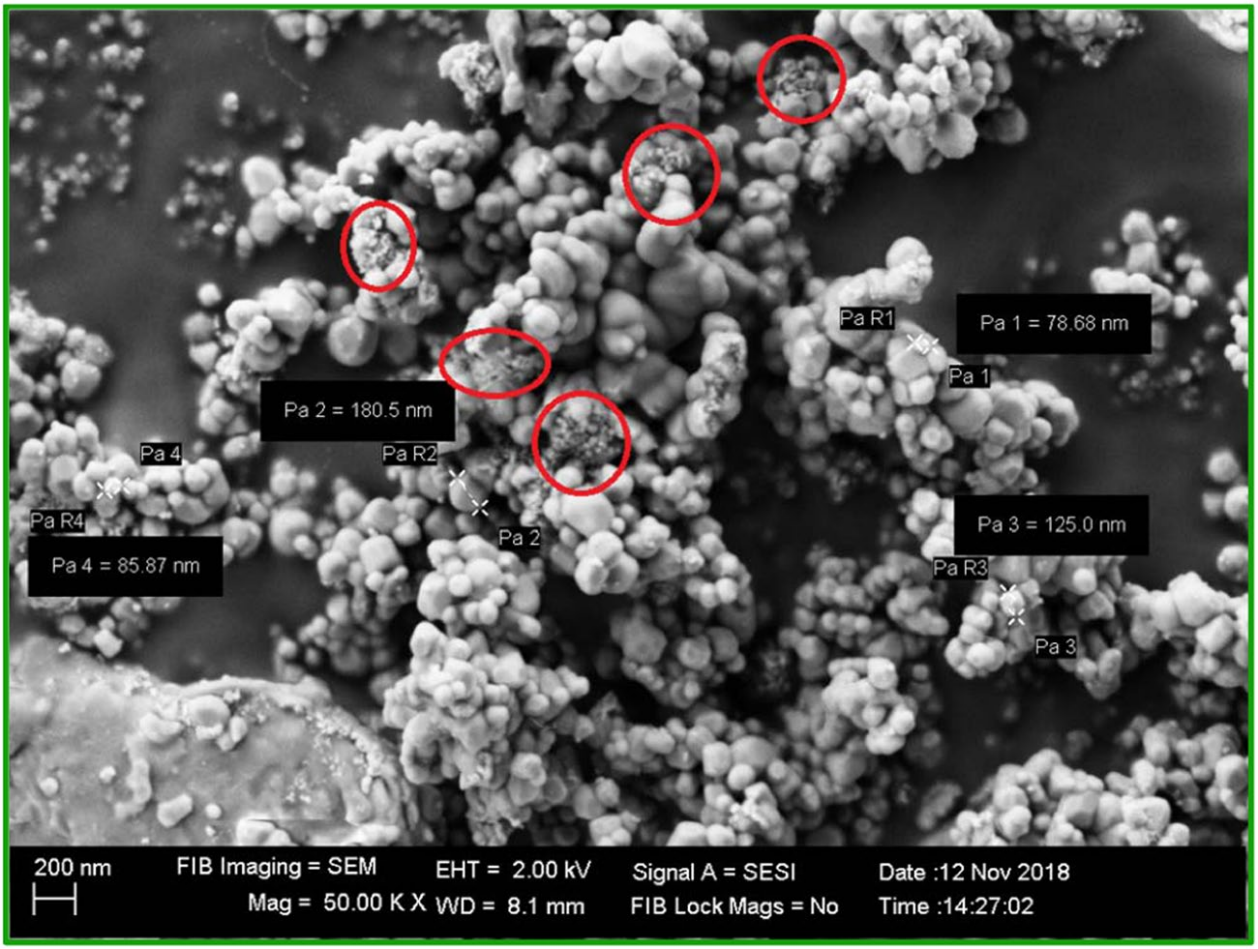
SEM image of TiO_2_-Fe_2_O_3_ nanoparticles.

These groups are possibly believed to influence the phase formation as well as in phase stabilization of all the nanocomposites^23–25^. The peak observed at460 cm^−1^ corresponding to Ti-O bond of TiO_2_, was found to be shifted to 525 cm^−1^ after the incorporation with Fe_2_O_3_where Fe-O stretching mode was clearly observed in the spectra. The peak at 1074 cm^−1^ is due to the C-O stretching vibration. The strong band below 700 cm^−1^ is assigned to Fe-O stretching mode. The band corresponding to Fe-O stretching mode of Fe_2_O_3_ is observed at 560 cm^−1^. Watchful observation of the SEM image of TiO_2_-Fe_2_O_3_ nanoparticles (Fig. 3) will reveal the presence of small Fe_2_O_3_ particles (circled in red color in Fig. 3) impregnated throughout the surface of TiO_2_ there by concluding that the larger particle is TiO_2_ and the smaller one is Fe_2_O_3_.

**Figure 4:**
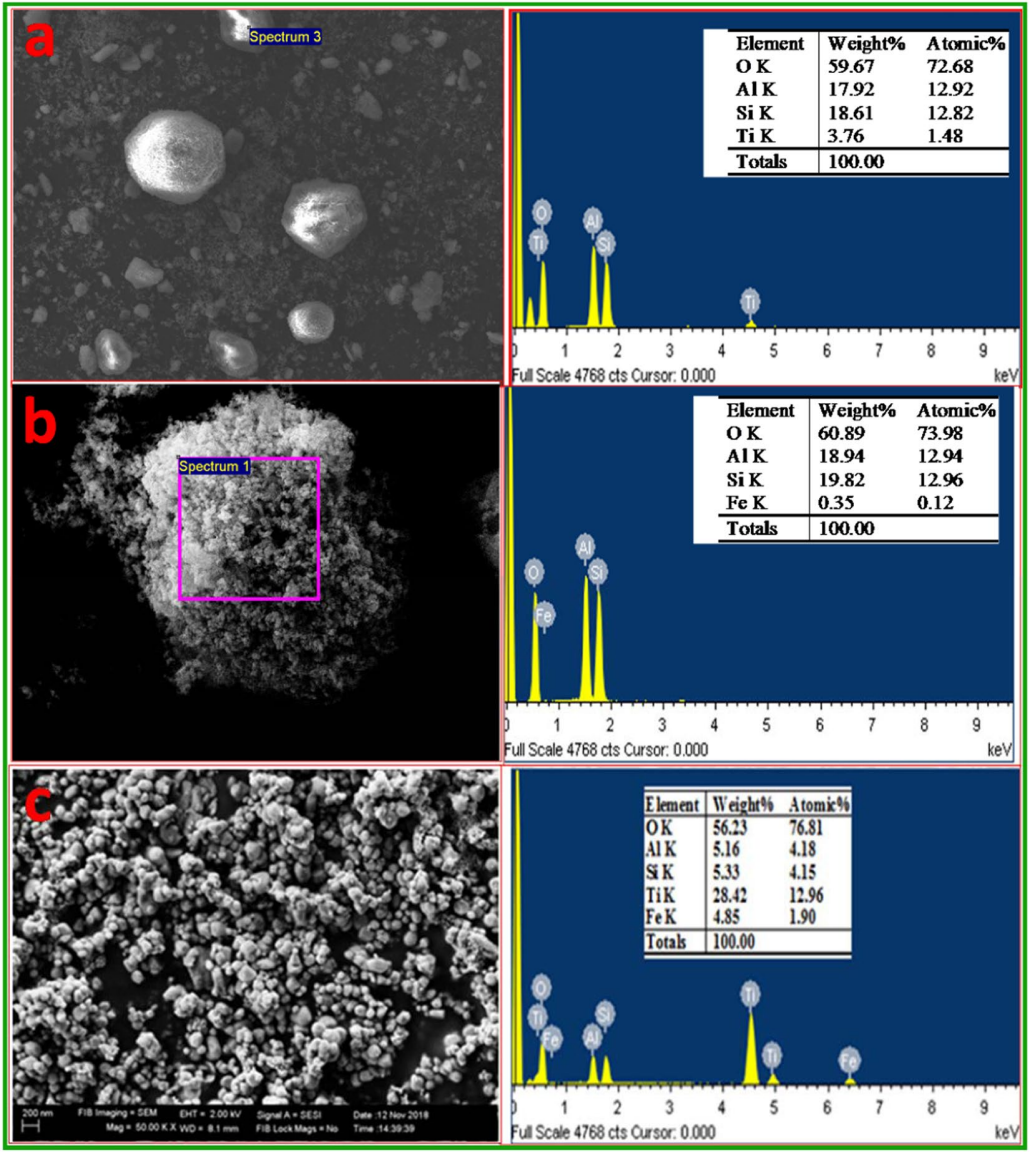
SEM images of (**a**) TiO_2_ with EDX, (**b**) Fe_2_O_3_ with EDX and (**c**) TiO_2_-Fe_2_O_3_ with EDX.

The SEM images of synthesized composites presented in Fig. 4(a–c) show that the morphology of the sample is somewhat porous and agglomerated, which is believed to be advantageous to enhance the properties. In addition, large and small particles of nearly equal sizes were observed in the images. The elemental confirmation in composites was done by using EDX measurements. The results revealed that the particle size of Fe_2_O_3_ is smaller than that of the TiO_2_ as observed in Fig. 4b. It is clear from the results that, increasing the calcination temperature causes the increase in the crystallinity of the material and helped to remove the impurities. Nevertheless, increasing the calcination temperature may also increase the crystal grain size^26,27^. In addition, the grain size which increases with surface roughness is believed to be advantageous in enhancing the contact surface area among electrode materials used in supercapacitors^28^.

**Figure 5:**
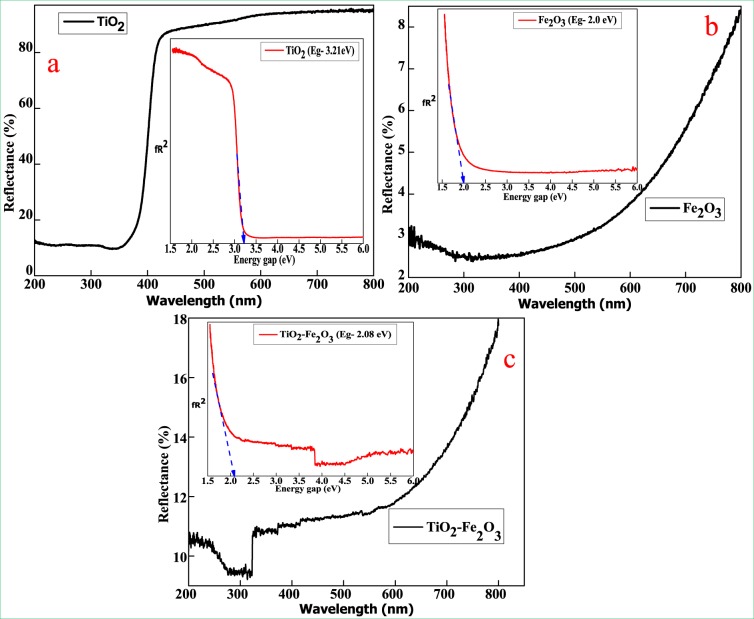
DRS and Energy Gap graphs of (**a**) TiO_2_, (**b**) Fe_2_O_3_ and (**c**) TiO_2_ - Fe_2_O_3_.

Figure 5(a–c) shows the UV-Visible diffused reflectance spectra of TiO_2_, Fe_2_O_3_ and TiO_2_-Fe_2_O_3_ nanomaterials. As shown in the spectra, TiO_2_ exhibit a strong light absorbance edge close to 380 nm due to its inherent band gap (~3.21 eV) presented in the inset of Fig. 5a, which matches with the band gap of TiO_2_-Fe_2_O_3_ showing absorbance edges close to 500 nm (Eg = 2.0 eV), which is in agreement with the band-gap energy of Fe_2_O_3_ presented in the inset of Fig. 5b,while, TiO_2_-Fe_2_O_3_ edge found between 490 nm and 700 nm can be attributed to TiO_2_ and Fe_2_O_3_, with band gap energy (E_g_ = 2.08 eV) as presented in the inset of Fig. 5c. This shows that, significant improvement of visible light reflection and thus improvement in electrochemical reactions by means of visible light in addition to ultra-violet^28,29^. As suggested by^30^, owing to unique half-filed electronic configuration, Fe has the capacity to form new energy levels within the band gap range of TiO_2_ and reduces the gap between valence band and conduction band (red-shift of the absorption threshold).

The Kubelka - Munk function F(R) is utilized for examining the powders as given by Eq. (1):

1 $${\rm{F}}({\rm{R}})=\frac{{(1-{\rm{R}})}^{2}}{2{\rm{R}}}$$

where R: the reflectance, F(R): Kubelka-Munk function.

The optical energy gap can be calculated by using Tauc relation; the band gap (E_g_) of semiconductor material can be calculated from the following equation:

2 $$F(R)h=A{({\rm{h}}-{\rm{Eg}})}^{n}$$

where n = 1/2 and 2 for direct and indirect transitions respectively, for the optical energy gap was calculated using Tauc relation as in Eq. (2) gives the direct band^31^.

### BET study

**Figure 6:**
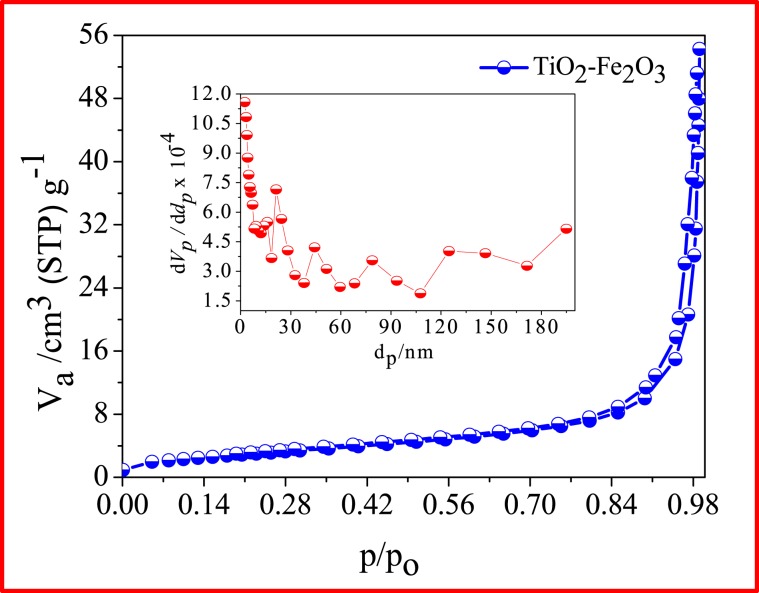
Adsorption-desorption measurements showing BJH plot (Inset- Pore size distribution).

The optimal porosity and high surface area (low particle size) are the two crucial things for the synthesized TiO_2_-Fe_2_O_3_ nanocomposite to be efficient catalyst. From Brunauer–Emmett–Teller (BET) study, N_2_ adsorption-desorption measurement carried out at 77 K (Fig. 6), the synthesized TiO_2_-Fe_2_O_3_ nanomaterial was found to exhibit type IV isotherm with a sharp increase of the adsorbed volume starting from P/P_0_ = 0.97. This confirms the presence of well-developed mesoporous nanostructured nature of nanomaterial. The shifting of the hysteresis loop to the higher as the relative pressure (p/p_o_) approaching to 1 indicates the presence of the micro porous particles with size greater than 50 nm. Furthermore, it also confirmed by the presence of mesoporous peaks around ~21, 44, 79, and 124 nm on the pore size distribution curve present as an inset in Fig. 6. The obtained BET surface area and pore volume value were found to be 71.56 m^2^/g and 0.076 cm^3^/g, respectively. The surface area of TiO_2_-Fe_2_O_3_ was found to be much higher relative to TiO_2_, which confirms the role of impregnated Fe_2_O_3_ in the enhancement of the specific surface area. From the equivalent, Barrett–Joyner–Halenda (BJH) pore-size, the obtained mean pore diameter for TiO_2_-Fe_2_O_3_ was found to be 2.43 nm.

### Photocatalytic studies of dye under UV light irradiation

A circular glass reactor with surface area of 176.6 cm^2^ was utilized for the dye decolorization studies. A 125 W medium pressure mercury vapor lamp was used as UV light source. The samples were irradiated directly by focusing light into the reaction mixture at a distance of 23 cm. In a distinctive experiment ~60 mg of photocatalyst was dispersed in 250 ml of 20 ppm different dye solutions. The reaction mixture was subjected to vigorous stirring using magnetic stirrer for the entire period of experiment. The extent of dye decolorization was calculated by using the equation, Q = (C_o_ − C)V/W,

where ‘Q’ is the extent of dye decolorization, C_o_ and C are the concentrations of dye before and after decolorization, Vis the volume of the reaction mixture and W is the mass of photo-catalyst present in grams. The unit of Q is ppm ml mg^−1^. 6 ml aliquots of solution were drawn from the suspensions at definite time intervals and centrifuged straight away and filtered through a what men filter paper.

### Decolorization of Titan Yellow (TY) and Methyl Orange (MO) dyes

**Figure 7:**
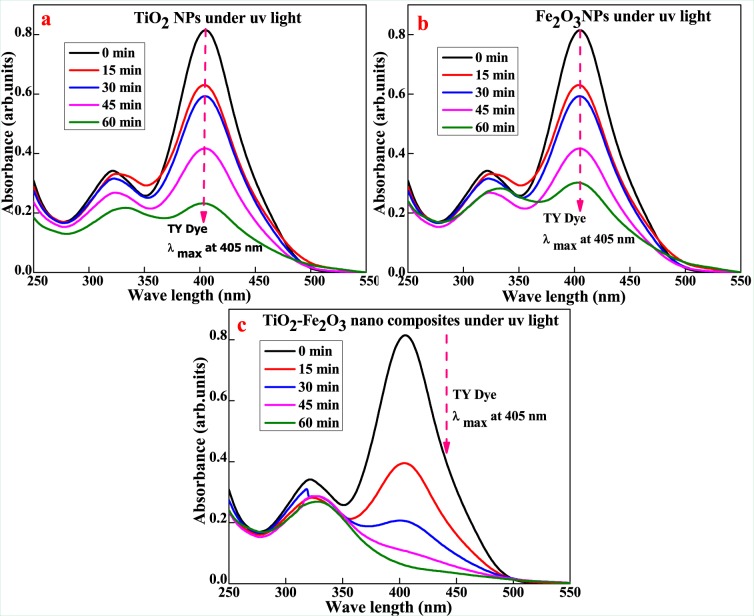
UV- Visible absorption spectra of TY dye (**a**) TiO_2_, (**b**) Fe_2_O_3_ and (**c**) TiO_2_ –Fe_2_O_3_under UV- light irradiation.

Photocatalytic studies of the synthesized TiO_2_, Fe_2_O_3_ and TiO_2_-Fe_2_O_3_ nanoparticles were analyzed to review the decolorization performance at room temperature for the degradation of Titan Yellow (TY) and Methyl Orange (MO) dyesin aqueous solution with concentration of 20 ppm under UV-light irradiation with 60 mg of optimum catalysts dosage, as shown in Fig. 7^32^.

**Figure 8:**
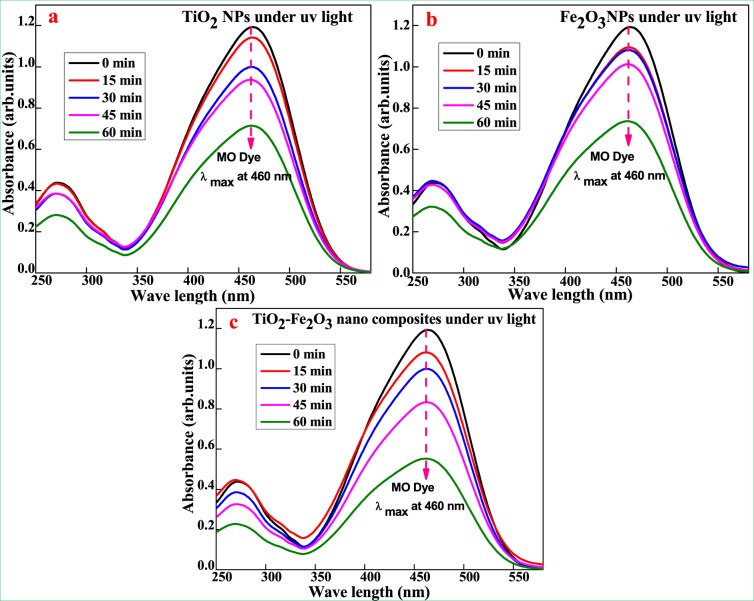
UV- Visible absorption spectra of MO dye (**a**) TiO_2_, (**b**) Fe_2_O_3_ and (**c**) TiO_2_ –Fe_2_O_3_under UV- light irradiation.

Photo-decolorization of TY dye for synthesized nanoparticles is shown in the Fig. 7(a–c) with evidence of UV-Visible absorption spectra. The TiO_2_-Fe_2_O_3_ nanocomposite was found to exhibitsuperior photo-decolorization results for TY dye solution when compared to TiO_2_ and Fe_2_O_3_ nanoparticles respectively. In the case of TY dye solution, the TiO_2_-Fe_2_O_3_ nanocomposite exhibited 92.98% dye decolorization as shown in Fig. 7c, which is superior performance compared to 71.67 and 62.89% for TiO_2_ and Fe_2_O_3_, respectively as revealed in Fig. 7a,b at 405 nm. Photocatalytic activity as presented in Fig. 7(a–c) revealed that the photo-decolorization effects in the presence of the TiO_2_-Fe_2_O_3_ nanocomposite were found to be more efficient and reached the maximum adsorption capability around 53.58%. Up to 60 min of UV light irradiation during the photocatalytic activity, the synthesized composite continued to exhibit the best decolorization capability with 53.58% decolorization of the MO dye solution as shown in Fig. 7c. TiO_2_ nanoparticles also exhibited good decolorization ability for the TY dye solution with a final removal of organic dye around 45.02% at 460 nm compared to decolorization ability of Fe_2_O_3_ nanoparticles. Fe_2_O_3_ nanoparticles ability to remove MO dye was found to be 38.67% as shown in Fig. 8^33–36^.

In addition to that, TiO_2_-Fe_2_O_3_ nanocomposite exhibited superior photocatalytic efficiency of 92.98% for the azo dye group under UV light irradiation for a period of 60 min. In the case of MO dye solutions, the TiO_2_-Fe_2_O_3_ composite exhibited very less photocatalytic activity of 53.58% after 60 minutes of irradiation which is found to bellower than that of TiO_2_ and Fe_2_O_3_ for MO organic dye after 60 minutes under UV light irradiation. From these results, it can be concluded that TiO_2_-Fe_2_O_3_ nanocomposite is an excellent photocatalyst for the decolorization of TY organic dye with high photo-decolorization activity which can be attributed to e^−^h^+^ recombination, generation of hydroxyl and superoxide radicals.

Rate constant k values for TiO_2_, Fe_2_O_3_ and TiO_2_–Fe_2_O_3_ were found to be 0.0194, 0.0159, 0.04396 for TY and 0.00931, 0.00772 and 0.0119 min^−1^ for MO dyes respectively. It clearly reveals that TiO_2_–Fe_2_O_3_ nanocomposite is better nanomaterial for TY dye showing enhanced photocatalytic activity compared to pure TiO_2_ and Fe_2_O_3_ synthesized nanoparticles. In addition, the noticeable rate constant k of the TiO_2_–Fe_2_O_3_ nanocomposite for MO dye is 0.44 times and 2.26 times to that of TiO_2_–Fe_2_O_3_nano-composite for TY dye respectively and rate constant can be ranked in the order of Fe_2_O_3_(MO) <TiO_2_(MO) <TiO_2_–Fe_2_O_3_(MO)<Fe_2_O_3_(TY) <TiO_2_(TY) <TiO_2_–Fe_2_O_3_(TY) was observed in Tables 1 and 2. High separation and transportation rate of the e^−^h^+^ pairs, and reduction of band gap are responsible for higher photocatalytic activity of TiO_2_–Fe_2_O_3_ nanocomposite which could be attributed to synthetic method and modified surface morphology of synthesized nanoparticles^37^.

**Table 1:** Rate parameters of (a) TiO_2_, (b) Fe_2_O_3_ and (c) TiO_2_-Fe_2_O_3_ under UV light for TY dye.

t	c	c/c0	log c/co	−log c/co	%D
**(a) 20PPM TY** + **60 mg TiO**_**2**_ + **UV**					
0	20	1	0	0	0
15	15.18	0.759	−0.11976	0.119758	24.1
30	14.507	0.72535	−0.13945	0.139452	27.465
45	10.192	0.5096	−0.29277	0.292771	49.04
60	5.665	0.28325	−0.54783	0.54783	71.675
			Slope	0.008458	
			Rate	**0.019478**	
**(b) 20PPM TY + 60 mg Fe**_**2**_**O**_**3**_** + UV**					
0	20	1	0	0	0
15	14.596	0.7298	−0.1368	0.136796	27.02
30	12.216	0.6108	−0.2141	0.214101	38.92
45	9.7487	0.487435	−0.31208	0.312083	51.2565
60	7.4211	0.371055	−0.43056	0.430562	62.8945
			Slope	0.006909	
			Rate	**0.015912**	
**(c) 20PPM TY** + **60 mg TiO**_**2**_**-Fe**_**2**_**O**_**3**_ + **UV**					
0	20	1	0	0	0
15	9.7536	0.48768	−0.31187	0.311865	51.232
30	5.0985	0.254925	−0.59359	0.593588	74.5075
45	2.709	0.13545	−0.86822	0.868221	86.455
60	1.4039	0.070195	−1.15369	1.153694	92.9805
			Slope	0.019092	
			Rate	**0.043968**

**Table 2:** Rate parameters of (a) TiO_2_, (b) Fe_2_O_3_ and (c) TiO_2_-Fe_2_O_3_ under UV light for MO dye.

t	c	c/c0	log c/co	−log c/co	%D
**(a) 20PPM MO** + **60 mg TiO**_**2**_ + **UV**					
0	20	1	0	0	0
15	19.1443	0.957215	−0.01899	0.018991	4.2785
30	16.694	0.8347	−0.07847	0.07847	16.53
45	15.671	0.78355	−0.10593	0.105933	21.645
60	10.996	0.5498	−0.2598	0.259795	45.02
			Slope	0.004044	
			Rate	**0.009312**	
**(b) 20PPM MO** + **60 mg Fe**_**2**_**O**_**3**_ + **UV**					
0	20	1	0	0	0
15	18.338	0.9169	−0.03768	0.037678	8.31
30	18.12	0.906	−0.04287	0.042872	9.4
45	15.302	0.7651	−0.11628	0.116282	23.49
60	12.265	0.61325	−0.21236	0.212362	38.675
			Slope	0.003356	
			Rate	**0.007728**	
**(c) 20PPM MO** + **60 mg TiO**_**2**_**-Fe**_**2**_**O**_**3**_ + **UV**					
0	20	1	0	0	0
15	18.1375	0.906875	−0.04245	0.042453	9.3125
30	16.744	0.8372	−0.07717	0.077171	16.28
45	13.9261	0.696305	−0.1572	0.1572	30.3695
60	9.2835	0.464175	−0.33332	0.333318	53.5825
			Slope	0.005209	
			Rate	**0.011997**	

Tables 1 and 2 presents kinetics parameters of TY and MO at various time periods (from 0 to 60 min) at 405 and 460 nm respectively, it evidently shows that the degradation of dye increased with time in the presence of synthesized nanoparticles under UV light irradiation^38^. TY dye decolorization of 92.98% for TiO_2_-Fe_2_O_3_ nanocomposite is very significant. From the log C/C_o_ values, it is also evident for decolorization of dyes under UV light irradiation time, exhibiting linear relationship based on the following equation:

3 $${\rm{logC}}/{\rm{Co}}=-\,{\rm{kt}}$$

where C_o_ is concentration of dye at time t = 0 min, C is a concentration of dye at particular time t and k is first order rate constant. This follows first order rate kinetics, photocatalytic decolorization efficiency of dye is calculated by using the following equation as efficiency (%) of photo-decolorization.

4 $$( \% {\rm{D}})=\frac{{{\rm{C}}}_{0}-{\rm{C}}}{{{\rm{C}}}_{0}\times 100}$$

where C_o_ is the initial concentration of the dye solution, C is a residual concentration of the dye in solution after degradation in equilibrium.

**Figure 9:**
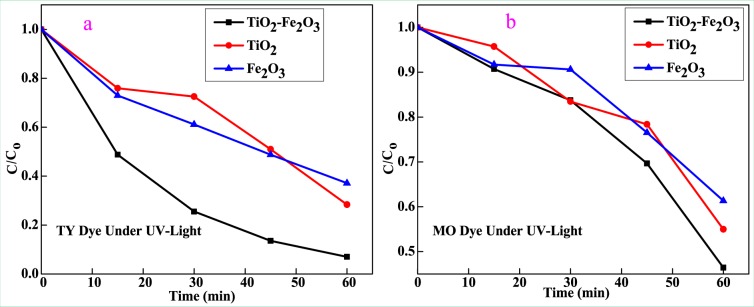
Plot of C/C_0_ for the decolorization of (**a**) TY dye and (**b**) MO dye under UV-Light illumination.

The photo-decolorization of dye in the presence of photocatalyst occurs as evidenced by little change in the absorption peak after UV light irradiated for 60 min^39^. Figure 9(a,b) showed plots of C/C_0_ for the decolorization of TY and MO, where C_o_ is initial concentration of dye and C is the concentrations of the dye at time t, respectively. It is undisputed that the decolorization rate of TY and MO followed by the order of Fe_2_O_3_(MO) <TiO_2_(MO) <TiO_2_-Fe_2_O_3_(MO)<Fe_2_O_3_(TY) <TiO_2_(TY) <TiO_2_-Fe_2_O_3_(TY) after the 60 min irradiation time. The result indicated that the decolorization efficiency of TY could be enhanced in the presence of TiO_2_-Fe_2_O_3_ nanocomposite photocatalytic system as compared to pure TiO_2_ and Fe_2_O_3_.

**Figure 10:**
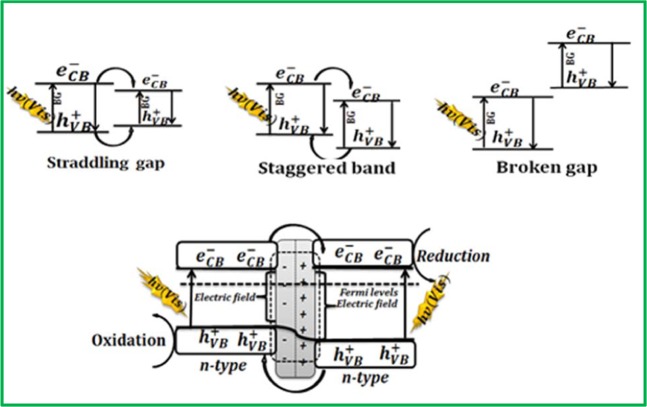
Schematic of depletion layer at the heterojunction with electron-hole pairs.

The electronic arrangement of the semiconductor heterojunctions is categorized into straddling gap, staggered gap, and broken gap. Among these, the staggered band structure has high probability in the direction of electron-hole (e^−^h^+^) separation and improving capacity of the photocatalytic reaction. This happens because of the creation of a depleted layer at the interface of the two (which produces an electric field) and then the diffusion of the photogenerated e^−^h^+^ out of the depletion layer takes places. Thus matching the band gaps of TiO_2_ and Fe_2_O_3_ play a significant role in the mechanism. During heterojunction, as shown in Fig. 10, when the Fermi level (FL) of one n-type semiconductor core in contact with the FL of the other n-type, the depletion layer that produces electric field was created.

**Figure 11:**
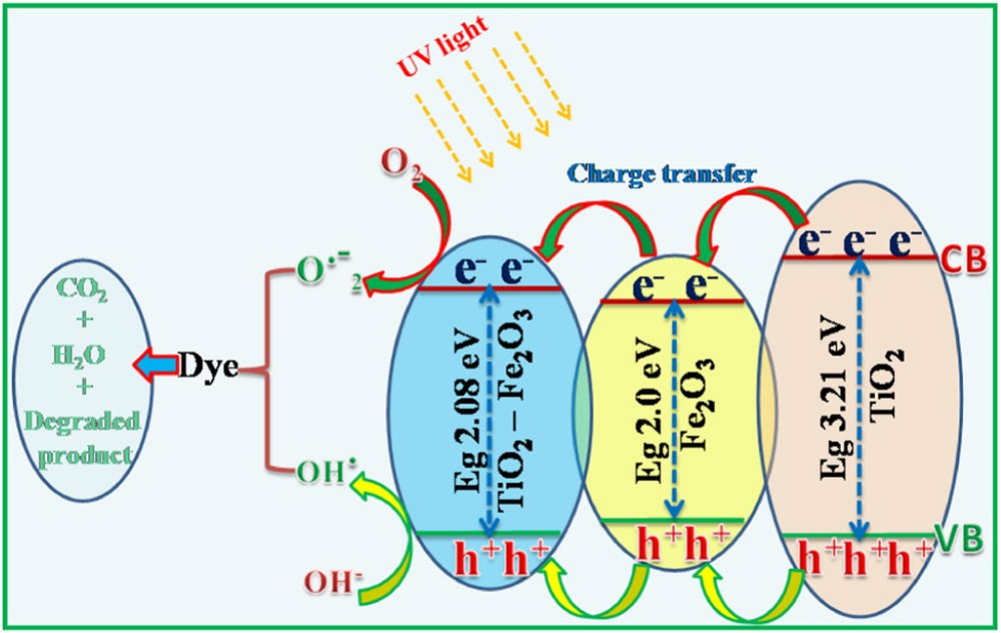
Schematic representation of energy band structure of TiO_2_, Fe_2_O_3_ and TiO_2_- Fe_2_O_3_ nano structures under UV light.

The activated electric field drives the diffusion of the photogenerated e–h+ out of the depletion layer and results in activation of the photocatalysis efficiency. In the higher energy region, some of the photogenerated e^−^s with a higher energy level than the CB position of TiO_2_ could thermodynamically transfer to the CB of TiO_2_ (Fig. 11), while photogenerated holes build up in the valence band of Fe_2_O_3_. The negatively charged e^−^s in the conduction band of TiO_2_ will further transfer to TiO_2_-Fe_2_O_3_ nanocomposite via Fe_2_O_3_^40^.

These negatively charged electrons, which are believed to react with O_2_ to form the superoxide anion (O^−^_2_) in the dye solution and hydrogen peroxide (H_2_O_2_), while positively charged holes are accompanied in the TiO_2_-Fe_2_O_3_ nanocomposite of the valence band, will respond with OH^−^groups present on the surface of the catalyst, produces reactive hydroxyl radicals (OH^.^). The reactions can be described as follows:

$${{\rm{Fe}}}^{3+}+{\rm{h}}v\to {{\rm{Fe}}}^{2+}+{{\rm{Fe}}}^{4+}+{{({\rm{h}}}_{{\rm{vb}}}}^{+}+{{\rm{e}}}_{{\rm{cb}}}^{\mbox{--}})$$

$${{\rm{Ti}}}^{4+}+{{{\rm{e}}}_{{\rm{cb}}}}^{-}\to {{\rm{Ti}}}^{3+}$$

$${{{\rm{e}}}_{{\rm{cb}}}}^{-}({{\rm{Ti}}}^{3+})+{{\rm{O}}}_{2}\to {{\rm{O}}}_{2}^{{\rm{\bullet }}}$$

$${{{\rm{h}}}_{{\rm{vb}}}}^{+}({{\rm{Fe}}}^{4+})+{{\rm{OH}}}^{-}{\to }^{{\rm{\bullet }}}{\rm{OH}}$$

$${{\rm{OH}}}^{{\rm{\bullet }}}+{\rm{dyes}}\to {\rm{degraded}}\,{\rm{products}}.$$

Thus, the enhanced photocatalytic decolorization effectiveness seen in the synthesized nano materials is basically attributed to more effective separation of photogenerated e^−^ and h^+^ pairs^41,42^.

## Electrochemical Studies

### Electrochemical behavior of the synthesized TiO_2_, Fe_2_O_3_ nanoparticles and TiO_2_-Fe_2_O_3_ nanocomposite

**Figure 12:**
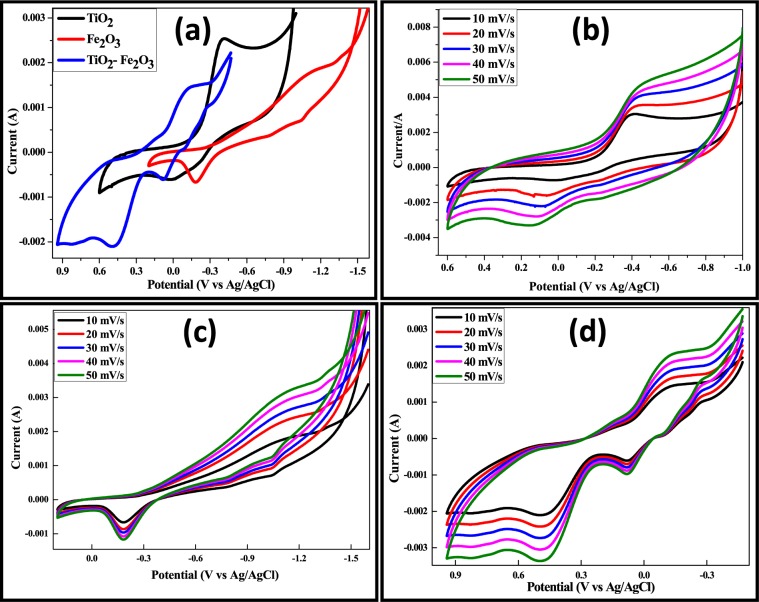
(**a**) CV of TiO_2_, Fe_2_O_3_ and TiO_2_-Fe_2_O_3_ electrode at 10 mVs^−1^. CV of (**b**) TiO_2_, (**c**) Fe_2_O_3_ and (**d**) TiO_2_-Fe_2_O_3_ electrode at various scan rates.

The appearance of oxidation peak during the charging and reduction peak during discharge process is usual for CV plots. CV was first performed in a range of −1.6 V and 0.9 at a scan rate of 10 mV/s as shown in Fig. 12.

The CV plot of TiO_2_ NPs shows one oxidation and reduction peak due to interconversion of Ti^2+^ and Ti^4+^, similarly same behavior can be seen in Fe_2_O_3_ NPs due to Fe^2+^ and Fe^4+^. But in the TiO_2_-Fe_2_O_3_ nanocomposite pair of oxidation-reduction peaks appears because of red-ox process of Ti-Fe composite. This behavior indicates that the capacity is mainly from the pseudo capacitance, which is based on the red-ox mechanism^43^.

For the nickel electrodes, the specific capacitance can be calculated from the CV curves, according to the following Eq. (4):

5 $${{\rm{C}}}_{{\rm{sp}}}=\frac{{\rm{i}}}{{\rm{r}}\times {\rm{m}}}$$

where i, r and m are the current, the scan rate and mass of the prepared TiO_2_, Fe_2_O_3_ and TiO_2_-Fe_2_O_3_ electrodes. The specific capacitance values were calculated to be 601 Fg^−1^, 385 Fg^−1^ and 879 Fg^−1^ for electrodes of TiO_2_, Fe_2_O_3_ and TiO_2_-Fe_2_O_3_ electrodes, respectively, at a scan rate of 10 mVs^−1^.

The relative heights of the peaks for each sample are found to be different. The observed discrepancy can be due to the difference in the effective surface area and amount of active material. The specific capacitance of electrodes was calculated by the following equation^43–45^:

6 $${\rm{C}}={\rm{i}}.\Delta {\rm{t}}/{\rm{m}}\Delta {\rm{V}}$$

where, *i* is the applied current, ΔV is the potential range, Δt is the time of a discharge cycle and m is the mass of the TiO_2_, Fe_2_O_3_ and TiO_2_-Fe_2_O_3_ nanocomposite. The specific capacitance values of pure TiO_2_, Fe_2_O_3_ and TiO_2_-Fe_2_O_3_ electrodes were calculated and found to be 585 Fg^−1^, 372 Fg^−1^ and 862 Fg^−1^. TiO_2_-Fe_2_O_3_ nanocomposite electrode exhibited highest capacitance.

**Figure 13:**
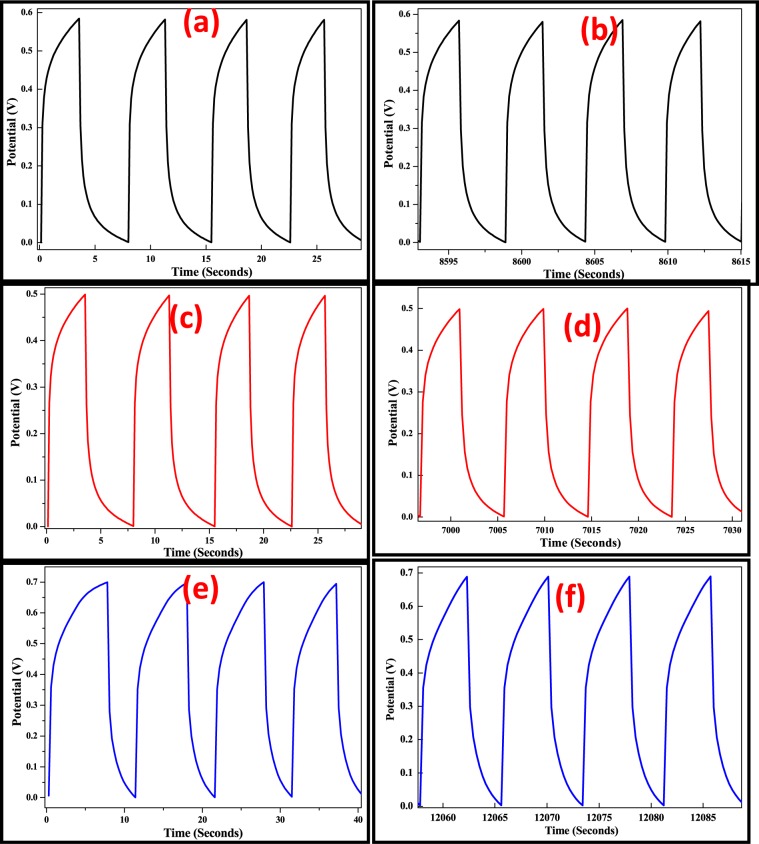
Galvanostatic Charge-Discharge curve of TiO_2_ (**a** and **b**), Fe_2_O_3_ (**b** and **c**) and TiO_2_- Fe_2_O_3_ nanocomposites electrodes (**e** and **f**) of 1^st^ and 1000^th^ cycle.

The life cycle stability of TiO_2_, Fe_2_O_3_ and TiO_2_-Fe_2_O_3_ electrodes were performed using galvanostatic charge-discharge curves (Fig. 13) measured at a current density of 5 Ag^−1^ for 1000 cycles within the potential window of 0–0.6 V (for TiO_2_), 0–0.5 V (for Fe_2_O_3_) and 0–0.7 V (for TiO_2_-Fe_2_O_3_) vs. Ag/AgCl. TiO_2_-Fe_2_O_3_ electrode exhibited very good stability after 1000 cycles.

**Figure 14:**
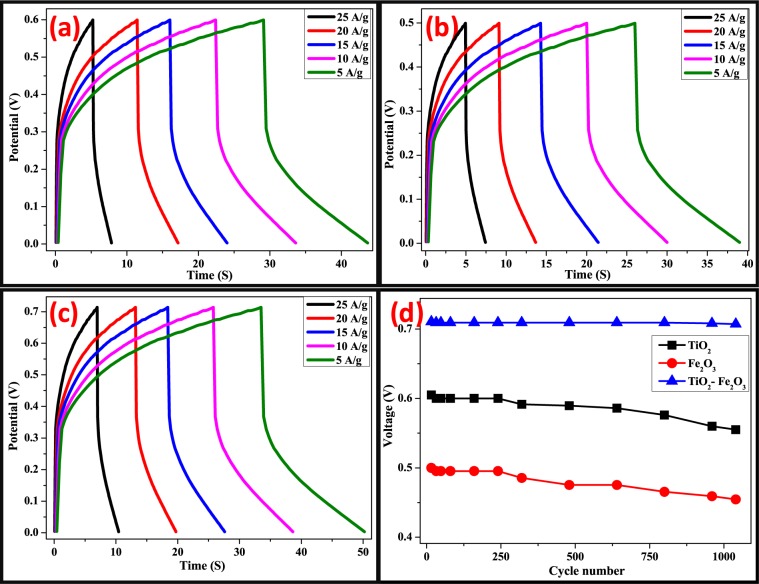
Galvanostatic charge-discharge curves of (**a**) TiO_2_, (**b**)Fe_2_O_3_ and(**c**) TiO_2_- Fe_2_O_3_ nanocomposite electrodes at various current densities. (**d**) Voltage as a function of Cycle number.

Galvanostatic charge–discharge curves of the TiO_2_, Fe_2_O_3_ and TiO_2_-Fe_2_O_3_ at various constant current densities are shown in Fig. 14. An ideal capacitive behavior is confirmed due to the appearance of triangular shaped curves in charge–discharge diagrams. The better storage rate ability of the synthesized materials was confirmed with an increase in integrated area on the current-potential axis^45^. The TiO_2_-Fe_2_O_3_ nanocomposite has the longest charge and discharge time, informative its maximum specific capacitance, which is in agreement with the consequences of Fig. 13, it confirms the voltage as a function of cycle number respectively revealing an opposite performance amongst them.

**Figure 15:**
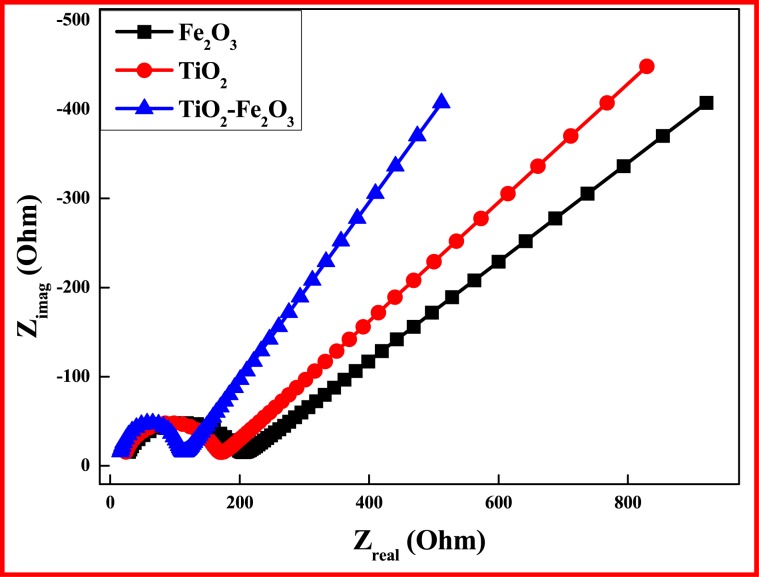
EIS spectra of TiO_2_, Fe_2_O_3_ and TiO_2_-Fe_2_O_3_ electrodes.

EIS measurements for TiO_2_, Fe_2_O_3_ and TiO_2_-Fe_2_O_3_ electrodes were carried out with three electrode assembly in 1 M KOH within the frequency range between 1 MHz and 100. The EIS spectra can be seen in Fig. 15. The real component Z_real_ reveals the ohmic properties while the imaginary part (Z_img_) relates to the capacitive properties^46^. Typically, semicircles with larger radii refer to higher charge transfer resistance of the electrodes^47^. Therefore, EIS result concludes that the charge transfer resistance R_ct_ of TiO_2_-Fe_2_O_3_electrode is much smaller than that of TiO_2_ and Fe_2_O_3_, indicating more effective incorporation of TiO_2_-Fe_2_O_3_nanostructures of this sample in the charge transfer process. Thus EIS results confirmed lower charge transfer resistance for TiO_2_-Fe_2_O_3_ electrode as compared to TiO_2_ and Fe_2_O_3_ electrode, and hence possess more capacitive properties^48^.

## Conclusions

We efficiently synthesized TiO_2_, Fe_2_O_3_ NPs and TiO_2_-Fe_2_O_3_ nanocomposites via green combustion route with Aloe Vera gel as a fuel. The diffraction peaks of TiO_2_ and Fe_2_O_3_ nanoparticles were well matched with tetragonal (anatase) and rhombohedral phasesas investigated by PXRD patterns. Furthermore, FTIR spectroscopy confirmed the formation of TiO_2_ and Fe_2_O_3_nanoparticles. The presence of the porous and agglomerated surfaces of the TiO_2_ as well as Fe_2_O_3_ and spherical for TiO_2_-Fe_2_O_3_ nanocomposites were observed through SEM and band gap energy of TiO_2_, Fe_2_O_3_ nanoparticles and TiO_2_-Fe_2_O_3_ nanocomposites were found to be 3.21, 2.0 and 2.08 eV respectively as confirmed by using DRS spectrum. The significant increase in the surface area of the nanocomposite as revealed by BET study confirmed the enhancement of adsorption capacity of pollutants and photocatalytic efficiency by impregnated TiO_2_-Fe_2_O_3_. The photo-decolorization studies revealed that TiO_2_-Fe_2_O_3_ nanocomposite is a good photocatalyst for the decolorization of Titan Yellow (TY) and Methyl Orange (MO)organic dyes with high photo degradation activity compared to TiO_2_ and Fe_2_O_3_ nanoparticles. The optimal conditions for this study included at room temperature in aqueous solution concentration of 20 ppm and catalyst dosage 60 mg under UV-light irradiation. The rate constants can be ranked in the order of Fe_2_O_3_(MO) < TiO_2_(MO) < TiO_2_–Fe_2_O_3_(MO) < Fe_2_O_3_(TY) < TiO_2_(TY) < TiO_2_–Fe_2_O_3_(TY). The synthesized TiO_2_-Fe_2_O_3_ nanocomposites showed excellent electrochemical behavior as electrode material for supercapacitance applications. A superior electrochemical response which includes enhanced charge/discharge capacity and cycling stability when compared to pure TiO_2_ and Fe_2_O_3_ nanoparticles resulted in stable electrochemical performance with nearly 100% coulombic efficiency at a high current density of 5 A/g for 1000 cycles. Interestingly, the novelty of this work is that the designed supercapacitors showed stable electrochemical performance even at 1000 cycles, which can be beneficial for rechargeable supercapacitor applications.
